# How do falls-risk increasing drugs modify the association between index falls and future falls and fracture burden? Secondary data analysis from the Irish longitudinal study on ageing

**DOI:** 10.1007/s41999-026-01549-0

**Published:** 2026-07-28

**Authors:** Thomas Cloney, Rose Anne Kenny, Robert Briggs

**Affiliations:** 1 Mercer’s Institute for Successful Ageing, St James’s Hospital, Dublin, Ireland; 2 Discipline of Medical Gerontology, Trinity College Dublin, Dublin, Ireland; 3 The Irish Longitudinal Study On Ageing, Trinity College Dublin, Dublin, Ireland

**Keywords:** Falls, Frailty, FRIDs, Fracture, Fragility Fracture

## Abstract

**Background:**

Falls are a major tipping point in older adults, heralding functional decline and increased healthcare utilisation. Falls-risk–increasing drugs (FRIDs) are a key risk factor for falls, but their influence on outcomes when prescribed after a fall is under-examined.

This study examined the association between an index fall requiring medical attention and subsequent 2-year risk of falls and fractures, evaluating how FRID use modifies this relationship.

**Methods:**

We analysed data from Waves 5 and 6 of The Irish Longitudinal Study on Ageing, including participants ≥ 70 years with 2-year follow-up.

FRIDs were identified from medication lists using STOPPFall.

Regression models were used to assess the association between index falls and falls/fracture outcomes during follow-up: Falls, falls requiring medical attention, emergency department visits due to falls, and fractures. Two-way interactions were used to assess how FRID use modified this association.

**Results:**

Of 1717 participants, 14% reported a fall requiring medical attention at baseline; 38% were prescribed ≥ 1 FRID.

Index falls were associated with increased future falls (OR 2.65), falls requiring medical attention (OR 3.82), ED attendance due to a fall (OR 2.93), and fractures (OR 2.46) over 2 years (all p < 0.01).

Combined exposure to both a fall and FRIDs further amplified risk, with odds ratios ranging from 3.77 to 4.36 across outcomes.

**Conclusion:**

A fall requiring medical attention is a strong predictor of future falls burden, and FRID use significantly amplifies this risk.

These findings highlight the need for targeted medication review within comprehensive geriatric assessment for older people with falls.

## Key summary points

**Aim:**

The primary aim of this study is to examine the association between a fall requiring medical attention and the subsequent 2-year burden of future falls and fractures in a large cohort of community-dwelling older people.

**Findings:**

Index falls were associated with increased future falls (OR 2.65), falls requiring medical attention (OR 3.82), ED attendance due to a fall (OR 2.93), and fractures (OR 2.46) over 2 years (all p0.01). Combined exposure to both a fall and FRIDs further amplified risk, with odds ratios ranging from 3.77 to 4.36 across outcomes.

**Message:**

An index fall requiring medical attention is a powerful predictor of future falls and fractures, with the risk significantly amplifi ed by concurrent use of FRIDs.

## Background

Falls represent a critical tipping point for older people, often leading to hospitalisation and marking the onset of serious decline in health and independence [1–3].

One in eight older people have a fall requiring medical attention annually, with 1 in 16 requiring an emergency department (ED) visit due to a fall [4]. Indeed falls, including injuries related to falls, can account for up to 20% of ED presentations in older people [5]. Notably, simple, low-impact falls are now the most common cause of major trauma in older adults, surpassing high-impact injuries like road traffic collisions [6]. This is especially significant given that older adults now represent the fastest-growing cohort of major trauma patients in most developed countries [6]. With increasing life expectancy and an ageing population, the burden of falls is expected to rise.

Secondary prevention of falls therefore represents a core component of comprehensive geriatric assessment (CGA), especially if an index fall was serious enough to warrant medical attention, and should address issues including gait and balance problems, cognitive impairment and orthostatic hypotension [7]. An important aspect of CGA focusing on falls prevention is medication reconciliation, as falls-risk increasing drugs (FRIDs) are a key modifiable risk factor for falls [8]. The Screening Tool of Older Persons Prescriptions in older adults with high fall risk (STOPPFall) can aid deprescribing by identifying medications that increase falls risk [9].

The medication classes identified by STOPPFall are benzodiazepines, antipsychotics, benzodiazepine-related drugs, opioids, antidepressants, antiepileptics, diuretics, alpha-blockers used as antihypertensives, alpha blockers used for prostate hyperplasia, centrally-acting antihypertensives, antihistamines, vasodilators used in cardiac diseases, and drugs for urinary frequency and incontinence [9]. These medications can cause falls by mechanisms including their effect on gait and balance, by causing sedation and cognitive impairment or by causing delayed orthostatic blood pressure recovery after standing [10–12].

Prescription of ≥ 2 FRIDs is independently associated with a significantly higher future burden of falls and falls-related injury, including a 60% higher likelihood of fracture [13]. Despite this, In Ireland, over one third of older people are prescribed at least a single FRID and almost three quarters admitted to hospital with a fall are prescribed FRIDs, with antidepressants and diuretics the most frequently prescribed [14].

While history of falls is an established risk factor for future falls [15], the specific longitudinal impact of a fall requiring medical attention, i.e., a fall requiring a visit to a doctor either in the community or in the acute hospital, on the future burden of falls and fractures remains under-examined, particularly concerning the effect of modifying risk factors. We specifically examined falls requiring medical attention (rather than any fall) because they should have afforded an opportunity for medical personnel intervene to prevent further falls, are generally more serious than ‘any falls’, and represent a burden to the healthcare system [16, 17].

Using data from the Irish Longitudinal Study on Ageing (TILDA), we have previously outlined the longitudinal association of STOPPFall medication use with future falls [13]. This research builds on this by specifically investigating how the use of FRIDs, identified via the STOPPFall tool, modifies future falls and fracture risk in older people following a fall.

The primary aim of this study therefore is to examine the association between a fall requiring medical attention and the subsequent 2-year burden of future falls and fractures in a large cohort of community-dwelling older people. Further, we will delineate how the use of FRIDs in this cohort modifies future falls and fracture risk related to an index fall. This analysis will clarify the potentially synergistic effect of a fall and ongoing FRID use on falls and fracture burden.

## Methodology

This study examines the relationship between an index fall requiring medical attention and future further falls and fractures over 2-year follow-up, and how this relationship is influenced by co-existing FRID use.

### Study design

Our study uses data from TILDA, combining both self-reported and clinically measured information to assess health status and healthcare access among older people in Ireland. Data collection occurs in waves every two years. This analysis includes data from Waves 5 and 6, which were conducted from 2018 to 2023.

TILDA recruitment at Wave 1 was conducted using a stratified clustered procedure. This involved random sampling postal addresses from the Irish Geo-Directory ensuring recruitment of a population-representative sample.

With a sample cohort of approximately 8000 + people, detailed information on all aspects of their lives is collected including: economic, health and social aspects [14].

In brief, data were collected through three main components:

1. Computer-assisted personal interviews conducted by social interviewers in participants' homes.
2. Self-completion questionnaires filled out by participants and returned by post.
3. Comprehensive health assessments performed at research centres or, when necessary, modified home-based assessments conducted by trained research nurses.

### Inclusion/exclusion criteria

**Fig. 1:**
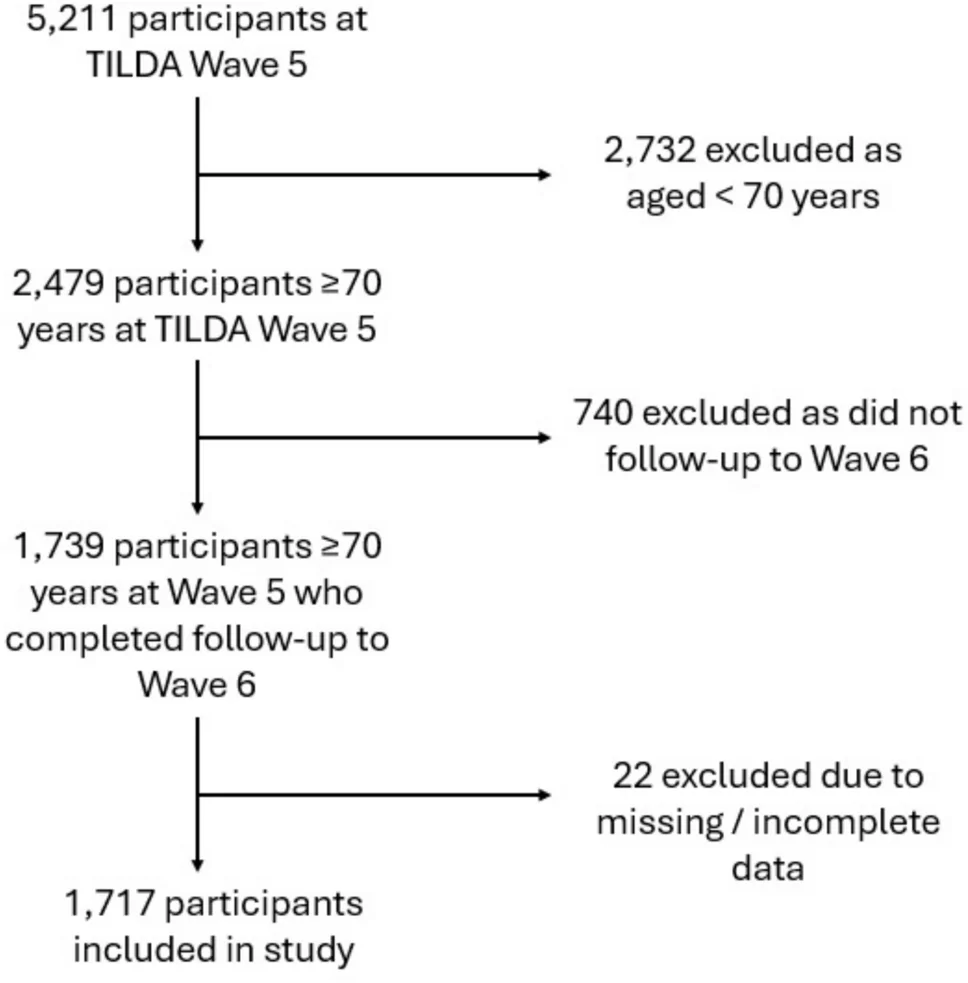
Flow Diagram of Study Recruitment

Participants were included in the study if they were aged ≥ 70 years at Wave 5, able to give informed consent to participate in the study, and completed the 2-year follow-up through to Wave 6. See Fig. 1 for Flow Diagram.

### Falls and fractures

At each wave (Waves 5 and 6), participants were asked, "Have you had any falls in the last year / since the last interview?" to assess fall incidence.

- If answered ‘yes’, participants were then asked if their fall was severe enough to required medical attention, which informed the ‘fall requiring medical attention’ variable. Medical attention comprises any visit to a doctor in community or hospital settings, including emergency department (ED) visits [4, 14].
- At follow-up interview at Wave 6, participants were asked if they had attended the ED since the last interview and if so, if this was due to a fall. This informed the ‘emergency department visit due to a fall’ variable.
- Participants were also asked about any fractures (hip, wrist, vertebral, or other) sustained since the last interview, i.e. between Waves 5 and 6. This information was used to create the "Fractures" variable.

### Falls risk-increasing drugs

Participant’s medication lists were examined, and medications recorded directly from this list, during the CAPI at Wave 5 (baseline) assessment, with medications identified by the Anatomical Therapeutic Chemical (ATC) Classification System.

FRIDs were identified using the STOPPFall tool [7], classifying the following medications as increasing the risk of falls: benzodiazepines (ATC codes N03AE, N05BA and N05CD), antipsychotics (ATC code N05A), benzodiazepine-related drugs (N05CF), opioids (N02A, R05DA), antidepressants (N06A, N06CA), antiepileptics (N03), diuretics (C02L, C03, C07B, C07C, C07D, C08GA, C09BA, C09BX01, C09BX03, C10BX13, C09DA, C09DX01, C09DX03, C09DX06, C09DX07, C09XA52, C09XA54), alpha-blockers used as antihypertensives (C02CA), alpha blockers used for prostate hyperplasia (G04CA), centrally-acting antihypertensives (C02A), antihistamines (N07CA02, R06), vasodilators used in cardiac diseases (C01D), and drugs for urinary frequency and incontinence (G04BD, G04CA53).

Anticholinergic medications were defined by the Anticholinergic Cognitive Burden (ACB) scale [18, 19] as those with ‘definite’ anticholinergic effects (i.e., a score of 3). Only anticholinergics not already classified in other STOPPFall classes were included to avoid duplication and included gastrological agents (A03AA07 A03AB05 A03BA01 A03BA03 A03BB01), Parkinsonian agents (N04AA01 N04AA02 N04AA04 N04AB02 N04AC01), antihistamines (N02BE51) and other medications (M03BA03 M03BC01 N05CM05 N05BB01).

### Covariates

Demographic and health-related covariates were assessed as follows:

- Educational attainment (primary, secondary, or tertiary) were self-reported.
- Alcohol intake was assessed using the CAGE scale [20].
- Polypharmacy was defined as being prescribed ≥ 5 medications.
- Heart disease was self-reported and included conditions such as heart attack, angina, congestive heart failure, and arrhythmia.
- Chronic disease burden was assessed based on self-reports of the following conditions: cancer, liver disease, Parkinson’s disease, osteoporosis, diabetes, eye diseases (e.g., glaucoma, cataracts, age-related macular degeneration), arthritis, and lung disease.
- Cognitive impairment was defined as a Mini Mental State Examination score < 25 [21].

### Statistical analysis

Data were analysed using Stata version 14.1 (StataCorp, Texas).

- Baseline characteristics were summarised for the study sample, stratified by fall status at Wave 5, presenting percentages with 95% confidence intervals.
- T-tests were used for continuous variables and Chi-Square for binary variables.
- We used regression models to assess the independent relationship between falls and outcomes, including:
  o Future falls
  o Future falls requiring medical attention
  o Future ED attendance due to a fall
  o Future fracture

All regression models were adjusted for age (70–74 years / 75–79 years/ ≥ 80 years), sex, educational attainment (primary / secondary / tertiary), alcohol excess (CAGE < 2 / CAGE ≥ 2 / Did Not Complete), chronic disease burden (none, one, or ≥ 2 conditions), heart disease, polypharmacy, and cognitive impairment. These covariates were selected a priori based on their potential to modify the relationship between falls and the outcomes of interest.

## Results

### Baseline characteristics

Of the 1,717 participants at Wave 5 (mean age 76 years, 54% female), 14% (95% CI 13%–16%) reported a fall requiring medical attention. As shown in Table 1, participants with falls were older and more likely to be female, with at least one chronic illness and had higher rates of polypharmacy.

**Table 1:** Baseline characteristics by falls

	Falls n = 237	Non-falls n = 1480	
Age (years), Mean (95% CI) Age breakdown: - 70–74 years - 75–79 years - ≥ 80 years	77.1 (76.5 – 77.8) 28% (23 – 34) 42% (36 – 48) 30% (24 – 36)	76.2 (75.9 – 76.5) 39% (36 – 41) 36% (34 – 39) 25% (23 – 27)	t = − 2.59; p = 0.009 X^2^ = 9.38; p = 0.009
Female, % (95% CI)	67% (60– 72)	52% (50 – 55)	X^2^ = 17.15; p < 0.001
Educational attainment, % (95% CI): - Primary - Secondary - Tertiary	31% (25 – 37) 33% (28 – 40) 36% (30 – 42)	26% (24 – 29) 38% (35 – 40) 36% (33 – 38)	X^2^ = 2.50; p = 0.286
CAGE Scale, % (95% CI): - CAGE < 2 - CAGE ≥ 2 - Did not complete	77% (71 – 82) 6% (3 – 9) 17% (13 – 23)	80% (78 – 82) 5% (4 – 6) 15% (14 – 17)	X^2^ = 0.75; p = 0.686
Polypharmacy, % (95% CI) ^A^	53% (46 – 59)	40% (38 – 43)	X^2^ = 13.19; p < 0.001
Heart disease, % (95% CI) ^B^	20% (16 – 26)	14% (12 – 16)	X^2^ = 6.66; p = 0.010
Chronic disease number, % (95% CI) ^C^ - None - One - Two or more	18% (14 – 24) 37% (31 – 43) 45% (38 – 51)	29% (27 – 31) 39% (36 – 41) 32% (30 – 35)	X^2^ = 17.41; p < 0.001
Cognitive impairment, % (95% CI) ^D^	7% (4 – 11)	5% (4 – 6)	X^2^ = 2.89; p = 0.089
FRIDs use ^E^	45% (39–52)	37% (34–39)	X^2^ = 6.42; p = 0.011

Baseline characteristics at Wave 5, by falls. Falls group reported falls requiring medical attention at baseline assessment. T-tests used for continuous variables, chi-square for binary variables

*CI* Confidence Interval, *CAGE* Cut Down, Angry, Guilty, Eye Opener, *FRIDs* Falls-Risk Increasing Drugs

A Polypharmacy defined as prescribed ≥ 5 medications; B Heart Disease defined as self-report of myocardial infarction, congestive cardiac failure, arrythmia or angina; C The Number of the following chronic diseases: cancer, liver disease, Parkinson’s disease, osteoporosis, diabetes, eye diseases (e.g., glaucoma, cataracts, age-related macular degeneration), arthritis, and lung disease; D Cognitive Impairment defined as MMSE < 25; E FRIDs assessed using STOPPFall

Over one third of participants were prescribed at least one FRID (38% (95% CI 35%–40%)) at baseline. The mean number of FRIDs prescribed per participant was 0.52 (95% CI 0.48 – 0.56), including 0.50 (95% CI 0.46 – 0.53) in those who did not report falls at baseline and 0.66 (95% CI 0.54 – 0.78) in those reporting falls requiring medical attention at baseline assessment (t = -2.91; p = 0.0037).

The rate of prescription of STOPPFall medication classes were as follows: 15% diuretics 15% in falls group, 15% in non-falls group), 9% antidepressants (14% in falls group, 8% in on-falls group), 7% alpha blockers used for prostate hyperplasia (8% in falls group, 6% in no-falls group), 5% opioids (8% in falls group, 4% in non-falls group), 4% benzodiazepines (6% in falls group, 4% in non-falls group), 3% benzodiazepine-related drugs (6% in falls group, 3% in non-falls group), 3% vasodilators used in cardiac diseases (3% in falls group, 3% in non-falls group), 2% antihistamines (1% in falls group, 2% in no-falls group), 2% alpha-blockers used as antihypertensives (1% in falls group, 2% in non-falls group), 1% antipsychotics (2% in falls group, 1% in no-falls group), 1% anticholinergics (2% in falls group, 1% in non-falls group), < 1% antiepileptics, centrally-acting antihypertensives and drugs for urinary frequency and incontinence.

### Future falls

Over half (54% (95% CI 48%–60%)) of participants with a fall requiring medical attention had a further fall during follow-up, compared to fewer than one third of those who did not report a fall at initial assessment (29% (95% CI 26%–31%)).

**Fig. 2:**
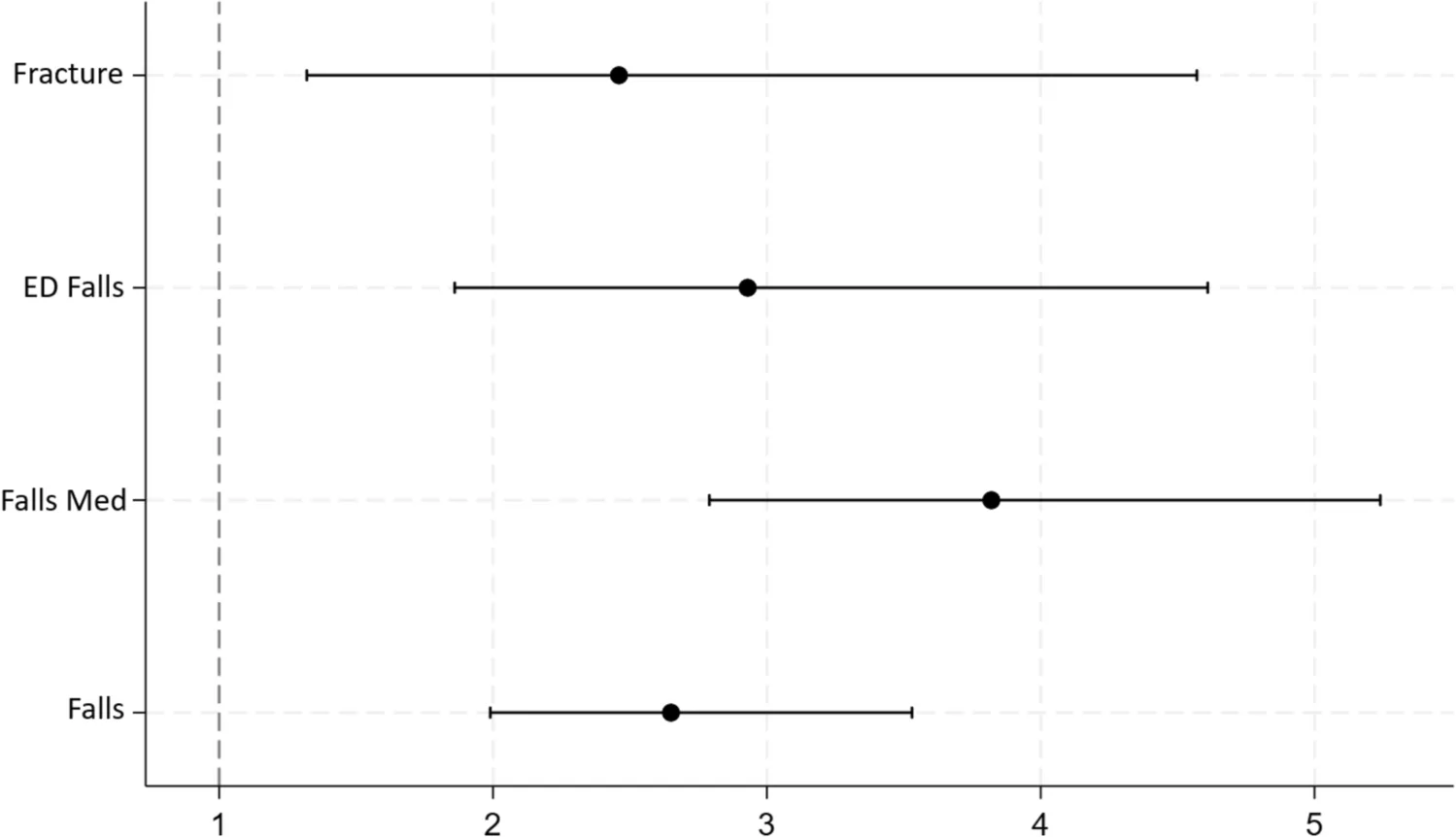
Adjusted Odds Ratios for Association of Falls Requiring Medical Attention with Future Falls and Fracture. Output from logistic regression model reporting odds ratios with 95% confidence intervals, for association of baseline falls requiring medical attention with the following longitudinal outcomes: falls, falls requiring medical attention, emergency department visit due to a fall, and fracture. Analysis adjusted for age, sex, educational attainment, alcohol excess, heart disease, polypharmacy, cognitive impairment and chronic disease burden. Follow-up: 2 years. N = 1717 (Falls at baseline, n = 237). Abbreviations: ED Falls = Emergency Department attendance due to falls during follow-up; Falls Med = Falls requiring medical attention during follow-up

In fully-adjusted models, a fall requiring medical attention was associated with a greater than 2.5-fold higher likelihood of future falls during 2-year follow-up (Odds Ratio 2.65 (95% CI 1.99 – 3.53); p < 0.001). See Fig. 2.

### Future falls requiring medical attention

Over one third (37% (95% CI 31%–43%) of participants with a fall requiring medical attention had a further fall requiring medical attention during 2-year follow-up, compared to 12% of those who did not report a fall at initial assessment (12% (95% CI 10%–14%).

In fully-adjusted models, a fall requiring medical attention was associated with an almost fourfold higher likelihood of a further fall requiring medical attention during 2-year follow-up (Odds Ratio 3.82 (95% CI 2.79 – 5.24); p < 0.001). See Fig. 2.

### Future emergency department visit due to falls

Over 14% (14% (95% CI 10%–19%)) of participants with a fall requiring medical attention had an emergency department (ED) visit due to a further fall in the following 2 years, compared to 5% (95% 4% -6%) of participants who did not report a fall at initial assessment.

As shown in Fig. 2, in fully-adjusted models, a fall requiring medical attention was associated with almost threefold higher likelihood of an ED visit due to falls during follow-u (Odds Ratio 2.93 (95% CI 1.86 – 4.61); p < 0.001).

### Future fracture

Almost 7% (7% (95% CI 4%–11%) of participants with a fall requiring medical attention had a fracture during follow-up, compared to 3% (95% CI 2%–3%) of those not reporting a fall at initial assessment.

In fully-adjusted models, a fall requiring medical attention was associated with a greater than twofold higher likelihood of fracture during follow-up (Odds Ratio 2.46 (95% CI 1.32 – 4.57); p = 0.008). See Fig. 2.

### Falls-risk increasing drugs

Almost half (45% (95% CI 39%–52%); n = 107/237) of participants reporting falls requiring medical attention were prescribed a FRID, compared to one third of those who did not report falls (37% (95% CI 34%–39%; n = 541/1,480).

In fully-adjusted two-way interaction models, the combination of falls requiring medical attention and FRID use was more strongly associated with future falls requiring medical attention (Odds Ratio 4.36 (95% CI 2.70 – 7.04; p < 0.001), future ED attendance due to a fall (Odds Ratio 3.77 (95% CI 1.94 – 7.34; p < 0.001) and future fracture (Odds Ratio 4.02 (95% CI 1.68 – 9.63); p = 0.002), than falls and/or FRID use in isolation. See Table 2.

**Table 2:** Fully-adjusted two-way interaction models for association of falls and FRIDs with future falls and fracture

Dependant variable: future falls requiring med attention	Odds ratio (95% CI)	p
Falls requiring medical attention # FRIDs - *Reference: No ‘Medical’ Falls, No FRIDs* - ‘Medical’ Falls, FRIDs + - ‘Medical’ Falls, No FRIDs - No ‘Medical’ Falls, FRIDs +	4.36 (2.69 – 7.04) 4.63 (3.02 – 7.10) 1.43 (1.01 – 2.03)	< 0.001 < 0.001 0.042
Dependant variable: future ED attendance due to falls	Odds ratio (95% CI)	p
Falls requiring medical attention # FRIDs - *Reference: No ‘Medical’ Falls, No FRIDs* - ‘Medical’ Falls, FRIDs + - ‘Medical’ Falls, No FRIDs - No ‘Medical’ Falls, FRIDs +	3.77 (1.94 – 7.34) 3.19 (1.67 – 6.11) 1.41 (0.83 – 2.39)	< 0.001 < 0.001 0.198
Dependant variable: future fracture	Odds ratio (95% CI)	p
Falls requiring medical attention # FRIDs - *Reference: No ‘Medical’ Falls, No FRIDs* - ‘Medical’ Falls, FRIDs + - ‘Medical’ Falls, No FRIDs - No ‘Medical’ Falls, FRIDs +	4.02 (1.68 – 9.63) 1.97 (0.76 – 5.06) 1.39 (0.68 – 2.84)	0.002 0.161 0.369

Output from logistic regression model reporting odds ratios with 95% confidence intervals, for association between 2-way interaction of falls and FRIDs at baseline with the following longitudinal outcomes: falls requiring medical attention, emergency department attendance due to falls, and fracture

Analysis adjusted for age, sex, educational attainment, alcohol excess, heart disease, polypharmacy, cognitive impairment and chronic disease burden

Follow-up: 2 years

*FRIDs* Falls-Risk Increasing Drugs by STOPPFALL criteria, ‘*Medical*’ *Falls* Falls requiring medical attention

## Discussion

This study demonstrates a clear association between an index fall requiring medical attention and the subsequent burden of falls and fractures. We found that a fall requiring medical attention independently predicts a two-fold increase in the likelihood of future fractures or additional falls, a three-fold increase in the risk of fall-related emergency department (ED) attendance, and a four-fold increase in the probability of another medically attended fall within two years. Importantly, our findings also show how the use of fall-risk–increasing drugs (FRIDs) after a fall further amplifies this future falls risk.

These findings are important as they underscore the need for evidence-based secondary prevention strategies following an initial fall. Although robust evidence indicates that multifactorial falls risk assessment can reduce monthly fall rates in older adults by nearly 30% [22], such interventions are not routinely incorporated into standard clinical practice, highlighting a persistent gap between research evidence and real-world care [23].

Building on previous evidence that FRIDs increase fall risk in older adults [13, 24], this study investigates the interplay between prior falls and FRID use to better predict future falls. Importantly, the risk of subsequent falls and fractures was substantially amplified by concurrent FRID use. The likelihood of sustaining a fracture during follow-up more than doubled when FRIDs were prescribed at the time of the index fall. This is particularly relevant given that over one third of community-dwelling older adults in Ireland, and nearly three quarters of older adults presenting to hospitals with falls, are prescribed at least one FRID [8, 14].

There are several mechanisms by which FRIDS may increase falls risk, and TILDA data has previously been used to demonstrate links between FRIDs and orthostatic hypotension, gait and balance problems and cognitive impairment [10, 12, 19], the ‘Bermuda Triangle’ of Falls [25].

Although deprescribing FRIDs can be challenging [26], our findings reinforce the need for systematic medication review, ideally embedded within fall-focused CGA, as a core component of secondary prevention, notwithstanding the need for robust randomised studies of deprescribing in the setting of falls [27].

There are some weaknesses of this study that should be noted. Falls are by self-report, though falls that require medical attention, that lead to an ED attendance or a fracture may be less subject to recall bias than all falls. Falls requiring medical attention also include falls resulting in an ED attendance. The risk of recall bias in the TILDA study is mitigated by use of informants, if deemed appropriate and with participants’ consent. Retrospective recall of falls (binary yes / no) has been shown to be relatively reliable in longitudinal studies [28]. Healthcare data on falls/fractures were unfortunately not available in this secondary data analysis. MMSE was used to define cognitive impairment as a covariate in the study. While rates of cognitive impairment were overall low, MMSE may misclassify come cases when used in the absence of direct adjustment for age and education and in the absence of a collateral history.

Strengths of the study include the recruitment of a large, well-described cohort of community-dwelling older people with relatively high prevalence of polypharmacy and chronic disease, followed for 2 years.

In conclusion, this study demonstrates that an index fall requiring medical attention is a powerful predictor of future falls and fractures, with the risk significantly amplified by concurrent use of FRIDs. Our findings have important clinical implications, underscoring the need for robust secondary prevention strategies, including targeted medication reviews, after an initial fall.

## References

[1] Howley F, Lavan A, Connolly E, McMahon G, Mehmood M, Briggs R (2021) Trends in emergency department use by older people during the COVID-19 pandemic. Eur Geriatr Med 12:1159–1167. 10.1007/S41999-021-00536-X PMC8285692 34273092

[2] Adam CE, Fitzpatrick AL, Leary CS, Ilango SD, Phelan EA, Semmens EO (2024) The impact of falls on activities of daily living in older adults: A retrospective cohort analysis. PLoS ONE 19(1):e0294017. 10.1371/journal.pone.0294017 PMC10763967 38170712

[3] Tinetti ME, Williams CS (1997) Falls, injuries due to falls, and the risk of admission to a nursing home. N Engl J Med 337(18):1279–1284. 10.1056/NEJM199710303371806 9345078

[4] Briggs R, Ward M, Scarlett S, van der Velde N, Hernandez B, Romero-Ortuno R, Tysinger B, May P, Ahern E, Kenny RA (2026) Determining the burden of falls amongst community-dwelling older people in Ireland to inform falls care delivery: secondary data analysis from the Irish longitudinal study on ageing - the defined study. BMJ Open 16(1):e107647. 10.1136/bmjopen-2025-107647 PMC12863362 41617230

[5] Jusmanova K, Rice C, Bourke R, Lavan A, McMahon CG, Cunningham C, Kenny RA, Briggs R (2021) Impact of a specialist service in the Emergency Department on admission, length of stay and readmission of patients presenting with falls, syncope and dizziness. QJM 114(1):32–38. 10.1093/qjmed/hcaa261 32866245

[6] Brent L, Hickey P, Brych O, Murray R. National Office of Clinical Audit (2024) Major Trauma Audit Report Focused on Older Adults 2017.

[7] Veronese N, Custodero C, Demurtas J, Smith L, Barbagallo M, Maggi S, Cella A, Vanacore N, Aprile PL, Ferrucci L, Pilotto A, Special Interest Group in Systematic Reviews of the European Geriatric Medicine Society (EuGMS); Special Interest Group in Meta-analyses and Comprehensive Geriatric Assessment of the European Geriatric Medicine Society (EuGMS) (2022) Comprehensive geriatric assessment in older people: an umbrella review of health outcomes. Age Ageing 51(5):afac104. 10.1093/ageing/afac104 35524746

[8] O’Donnell D, Zainal T, Malomo K, Fitzpatrick N, Rice C, Byrne L, Briggs R, Cunningham C, Kenny RA, Lavan AH (2025) Inpatient referrals to a specialist falls and syncope service: prevalence of STOPPFall FRIDs and review of deprescribing patterns. Eur J Clin Pharmacol 81(2):291–299. 10.1007/s00228-024-03776-5 39578287

[9] Seppala LJ, Petrovic M, Ryg J, Bahat G, Topinkova E, Szczerbińska K, van der Cammen TJM, Hartikainen S, Ilhan B, Landi F, Morrissey Y, Mair A, Gutiérrez-Valencia M, Emmelot-Vonk MH, Mora MÁC, Denkinger M, Crome P, Jackson SHD, Correa-Pérez A, Knol W, Soulis G, Gudmundsson A, Ziere G, Wehling M, O’Mahony D, Cherubini A, van der Velde N (2021) STOPPFall (screening tool of older persons prescriptions in older adults with high fall risk): a Delphi study by the EuGMS task and finish group on fall-risk-increasing drugs. Age Ageing 50(4):1189–1199. 10.1093/ageing/afaa249 PMC8244563 33349863

[10] O’Donnell D, Moriarty F, Lavan A, Kenny RA, Briggs R (2025) The association between psychotropic medication use and gait and mobility impairment in community-dwelling older people: data from the irish longitudinal study on ageing (TILDA). J Gerontol A Biol Sci Med Sci. 80(5):glae263. 10.1093/gerona/glae263 PMC12036327 39501425

[11] Chandramouleeshwaran S, Khan WU, Inglis F, Rajji TK (2024) Impact of psychotropic medications on cognition among older adults: a systematic review. Int Psychogeriatr 36(12):1110–1127. 10.1017/S1041610223000844 37860872

[12] Doyle K, Scarlett S, Moriarty F, Lavan A, Kenny RA, Briggs R (2025) The association between STOPPFall medication use and orthostatic hypotension in community-dwelling older people. Age Ageing 54(12):afaf347. 10.1093/ageing/afaf347 PMC12684716 41359604

[13] Doyle K, Scarlett S, Knight SP, Moriarty F, Lavan A, Kenny RA, Briggs R (2025) The association between STOPPFall medication use and falls and fractures in community-dwelling older people. Age Ageing 54(5):afaf138. 10.1093/ageing/afaf138 PMC12120935 40439666

[14] O'Connor D, Hever A, Scarlett S, Kenny RA. TILDA Wave 6 Report: Shaping the Future: Longitudinal trends and opportunities for transformation in health and social care in Ireland. 10.38018/TildaRe.2025-01

[15] Li Y, Hou L, Zhao H, Xie R, Yi Y, Ding X (2023) Risk factors for falls among community-dwelling older adults: a systematic review and meta-analysis. Front Med (Lausanne) 6(9):1019094. 10.3389/fmed.2022.1019094 PMC9853191 36687461

[16] Newgard CD, Lin A, Caughey AB, McConnell KJ, Bulger E, Malveau S, Staudenmayer K, Griffiths D, Eckstrom E (2022) Falls in older adults requiring emergency services: mortality, use of healthcare resources, and prognostication to one year. West J Emerg Med 23(3):375–385. 10.5811/westjem.2021.11.54327 PMC9183773 35679504

[17] Newgard CD, Lin A, Caughey AB, Eckstrom E, Bulger EM, Staudenmayer K, Maughan B, Malveau S, Griffiths D, McConnell KJ (2021) The cost of a fall among older adults requiring emergency services. J Am Geriatr Soc 69(2):389–398. 10.1111/jgs.16863 33047305

[18] Lisibach A, Benelli V, Ceppi MG, Waldner-Knogler K, Csajka C, Lutters M (2021) Quality of anticholinergic burden scales and their impact on clinical outcomes: a systematic review. Eur J Clin Pharmacol 77(2):147–162. 10.1007/s00228-020-02994-x PMC7803697 33011824

[19] Moriarty F, Savva GM, Grossi CM, Bennett K, Fox C, Maidment I, Loke YK, Steel N, Kenny RA, Richardson K (2021) Cognitive decline associated with anticholinergics, benzodiazepines and Z-drugs: findings from The Irish Longitudinal Study on Ageing (TILDA). Br J Clin Pharmacol 87(7):2818–2829. 10.1111/bcp.14687 33270264

[20] Bush B, Shaw S, Cleary P, Delbanco TL, Aronson MD (1987) Screening for alcohol abuse using the CAGE questionnaire. Am J Med 82(2):231–235. 10.1016/0002-9343(87)90061-1 2880504

[21] Folstein MF, Folstein SE, McHugh PR (1975) “Mini-mental state”. A practical method for grading the cognitive state of patients for the clinician. J Psychiatr Res 12(3):189–198. 10.1016/0022-3956(75)90026-6 1202204

[22] Chang JT, Morton SC, Rubenstein LZ, Mojica WA, Maglione M, Suttorp MJ, Roth EA, Shekelle PG (2004) Interventions for the prevention of falls in older adults: systematic review and meta-analysis of randomised clinical trials. BMJ 328(7441):680. 10.1136/bmj.328.7441.680 PMC381224 15031239

[23] Alvarado N, McVey L, Wright J, Healey F, Dowding D, Cheong VL, Gardner P, Hardiker N, Lynch A, Zaman H, Smith H, Randell R (2023) Exploring variation in implementation of multifactorial falls risk assessment and tailored interventions: a realist review. BMC Geriatr 23(1):381. 10.1186/s12877-023-04045-3 PMC10286425 37344760

[24] Zia A, Kamaruzzaman SB, Tan MP (2017) The consumption of two or more fall risk-increasing drugs rather than polypharmacy is associated with falls. Geriatr Gerontol Int 17(3):463–470. 10.1111/ggi.12741 26822931

[25] Donnell DO, Romero-Ortuno R, Kennelly SP, O’Neill D, Donoghue PO, Lavan A, Cunningham C, McElwaine P, Kenny RA, Briggs R (2023) The “Bermuda Triangle” of orthostatic hypotension, cognitive impairment and reduced mobility: prospective associations with falls and fractures in The Irish Longitudinal Study on Ageing. Age Ageing 52(2):afad005. 10.1093/ageing/afad005 PMC9897301 36735845

[26] Kalim RA, Cunningham CJ, Ryder SA, McMahon NM (2022) Deprescribing medications that increase the risk of falls in older people: exploring doctors’ perspectives using the theoretical domains framework (TDF). Drugs Aging 39(12):935–947. 10.1007/s40266-022-00985-4 PMC9676799 36409404

[27] Lee J, Negm A, Peters R, Wong EKC, Holbrook A (2021) Deprescribing fall-risk increasing drugs (FRIDs) for the prevention of falls and fall-related complications: a systematic review and meta-analysis. BMJ Open 11(2):e035978. 10.1136/bmjopen-2019-035978 PMC7878138 33568364

[28] Mackenzie L, Byles J, D’Este C (2006) Validation of self-reported fall events in intervention studies. Clin Rehabil 20(4):331–339. 10.1191/0269215506cr947oa 16719031

